# Catch-and-Release of Target Cells Using Aptamer-Conjugated Electroactive Zwitterionic Oligopeptide SAM

**DOI:** 10.1038/srep43375

**Published:** 2017-03-07

**Authors:** Junko Enomoto, Tatsuto Kageyama, Tatsuya Osaki, Flavia Bonalumi, Francesca Marchese, Alfonso Gautieri, Elena Bianchi, Gabriele Dubini, Chiara Arrigoni, Matteo Moretti, Junji Fukuda

**Affiliations:** 1 Graduate School of Engineering, Yokohama National University, Japan; 2 Department of Electronics, Information and Bioengineering, Politecnico di Milano, Italy; 3 Department of Chemistry, Politecnico di Milan, Italy; 4 Cell and Tissue Engineering Laboratory, IRCCS Galeazzi Orthopaedic Institute, Italy; 5 Regenerative Medicine Technologies Lab, Ente Ospedaliero Cantonale, Switzerland; 6 Swiss Institute for Regenerative Medicine, Switzerland; 7 Cardiocentro Ticino, Switzerland

## Abstract

Nucleic acid aptamers possess attractive features such as specific molecular recognition, high-affinity binding, and rapid acquisition and replication, which could be feasible components for separating specific cells from other cell types. This study demonstrates that aptamers conjugated to an oligopeptide self-assembled monolayer (SAM) can be used to selectively trap human hepatic cancer cells from cell mixtures containing normal human hepatocytes or human fibroblasts. Molecular dynamics calculations have been performed to understand how the configurations of the aptamers are related to the experimental results of selective cell capture. We further demonstrate that the captured hepatic cancer cells can be detached and collected along with electrochemical desorption of the oligopeptide SAM, and by repeating these catch-and-release processes, target cells can be enriched. This combination of capture with aptamers and detachment with electrochemical reactions is a promising tool in various research fields ranging from basic cancer research to tissue engineering applications.

Isolation of target cells from a mixture is fundamental in various research fields associated with cell culture including molecular cell biology, cancer research, and regenerative medicine. Several approaches have been employed to harvest target cells such as cancer cells, parenchymal cells, and stem or differentiated cells for subsequent culture and analysis^1^,^2^,^3^. Conventional approaches include density-gradient centrifugation and size-dependent microfluidic filtration^4^. However, a major limitation of these approaches is the lack of specificity to target cells because of overlaps of cell densities and sizes between the targets and other cells. Fluorescent- and magnetic-activated cell sorting methods have therefore emerged in the past decades and are now commercially available. Antibodies against cell surface molecules are responsible for the specificity to target cells in these approaches. A potential drawback of the use of antibodies is that specific membrane proteins and their arrangement need to be previously elucidated to acquire antibodies against target cells.

Nucleic acid aptamers provide an attractive and practical alternative^5^,^6^. Without prior knowledge of distinct marker proteins, aptamers against specific cells can be selected from pools of random-sequence oligonucleotides by means of so-called cell-SELEX (cell-systematic evolution of ligands by exponential enrichment)^7^,^8^. Aptamers bind to marker molecules with high specificity and affinity comparable to those of antibodies. In addition, aptamers possess several advantages over typical antibodies including rapid (<1 week) *in vitro* acquisition and replication^9^,^10^, stable long-term storage as a powder or solution, and easy and controllable modification^11^,^12^. Several reports have already shown that aptamers can be used for capturing target cells and subsequently releasing them for following analysis^13^. For example, lymphoblasts were captured on an aptamer-displayed substrate and then released by dissociating aptamer-cell binding with complementary nucleic acids^14^. Although the feasibility of the aptamer-based approach has been primarily examined for non-adherent cell populations such as blood cells, its adaptation to adherent cell populations still lags behind. This is probably because surfaces need to be more precisely designed to prevent random cell adhesion. To the best of our knowledge, there is no report showing the isolation and collection of adherent cells from other adherent cells using an aptamer-modified substrate.

The present study describes a design of culture substrates covered with an aptamer-conjugated oligopeptide layer for the isolation of adherent cells, using molecular dynamics (MD) calculations and experiments. In the cell isolation, a cell-repulsive background is responsible for preventing non-specific protein adsorption and thus non-specific cell adhesion. To this end, we employed zwitterionic oligopeptides that formed a dense self-assembled monolayer (SAM) on a gold substrate and served as a cell-repulsive background^15^. Another critical factor for the specific cell capture is the difference in binding affinity to an aptamer between target and non-target cells. We investigated the configurations of an aptamer in bulk and after conjugation to the oligopeptide SAM using MD calculations, because these could be responsible for the affinity. Electrochemical reactions have been used to desorb the SAM by cleaving the bond between the SAM and gold^16^,^17^. We hypothesized that target cells captured on the aptamer-conjugated SAM can be collected by applying an electrochemical potential (Fig. 1). Using human hepatic cancer cells as a target and normal human hepatocyte or human fibroblasts as non-targets, we examined whether the aptamer-conjugated surface can be used to selectively capture and then release the target cells without sever cytotoxicity.

## Results and Discussion

### Conformation of aptamer in bulk

A single-stranded DNA aptamer that specifically recognizes hepatic cancer cells was selected for the present study. The sequence of the aptamer is 5′- TAACTCAATAAGCTAGGTGGGTGGGGGACACTACTCGGGGGTGGTTGGGT-3′, which was previously obtained by means of cell-SELEX using human hepatoma Hep G2 cells as targets and human normal hepatocytes as counterparts^18^. Here we calculated the conformations of the single aptamer in explicit water, showing that a part of the aptamer (nucleotides GUA27 to THY18) forms a handle structure that is maintained until the end of the simulation (Fig. 2A). This region is similar to that predicted previously using the mfold web server^18^. We further tested the approximation of the implicit solvent, showing that it leads to similar results comparable to that in an explicit solvent. In particular, the end-to-end distance (139.47 ± 9.50 Å in explicit, 111.05 ± 19.27 Å in implicit), the radius of gyration (44.30 ± 0.82 Å in explicit, 38.81 ± 3.37 Å in implicit), and the persistence length (42.35 ± 1.88 Å in explicit, 43.54 ± 5.50 Å in implicit) showed no statistically significant difference. Subsequent simulations were therefore conducted in an implicit solvent.

### Behavior of aptamer on oligopeptide SAM

**Figure 1:**
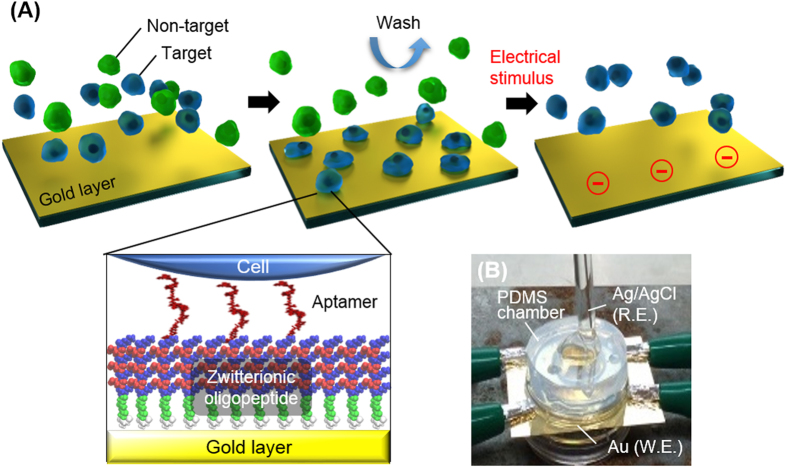
Concept of catch-and-release of target cells. (**A**) Procedures for selective capture and subsequent detachment of target cells using a surface modified with the zwitterionic oligopeptide SAM and the aptamer. (**B**) Setup of the two-electrode system consisting of a gold working electrode (W.E.) and a Ag/AgCl reference electrode (R.E.). The gold substrate was connected to four clips to alleviate a voltage drop.

**Figure 2:**
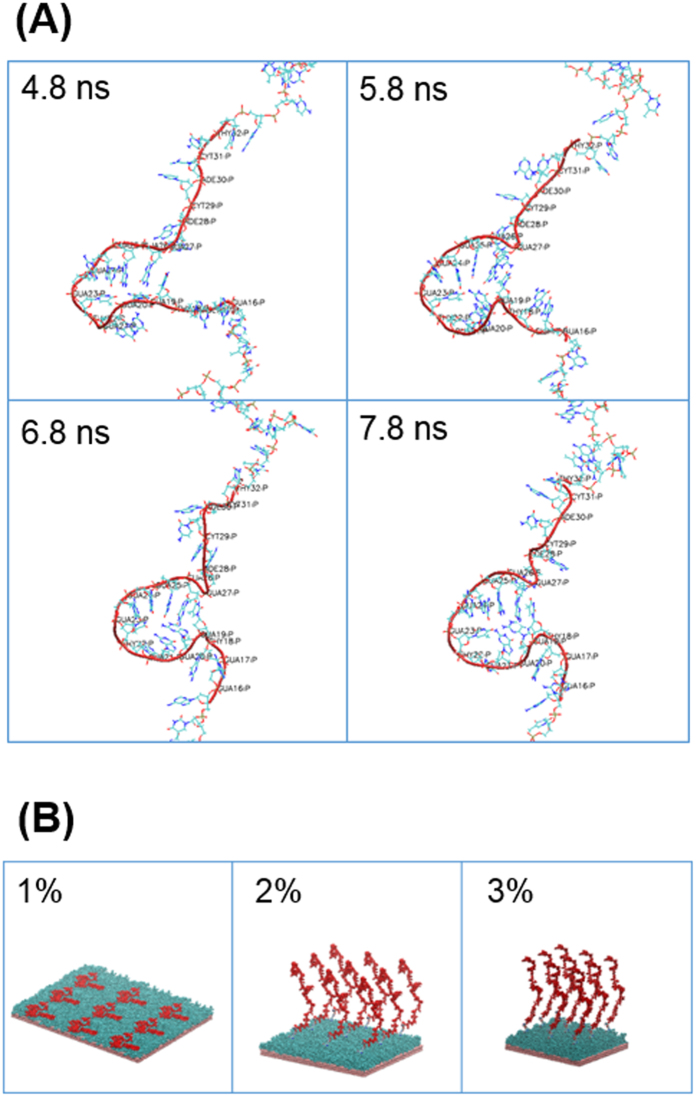
MD simulations of aptamer in bulk and on oligopeptide SAM. (**A**) Changes in aptamer configuration in explicit solvent in bulk. The values indicate periods of MD calculation. (**B**) Aptamer configuration on the oligopeptide SAM in implicit solvent. The values indicate the aptamer density (aptamer/oligopeptide). Gold layers (pink), oligopeptide SAMs (cyan), and aptamers (red) were visualized by VMD software.

**Figure 3:**
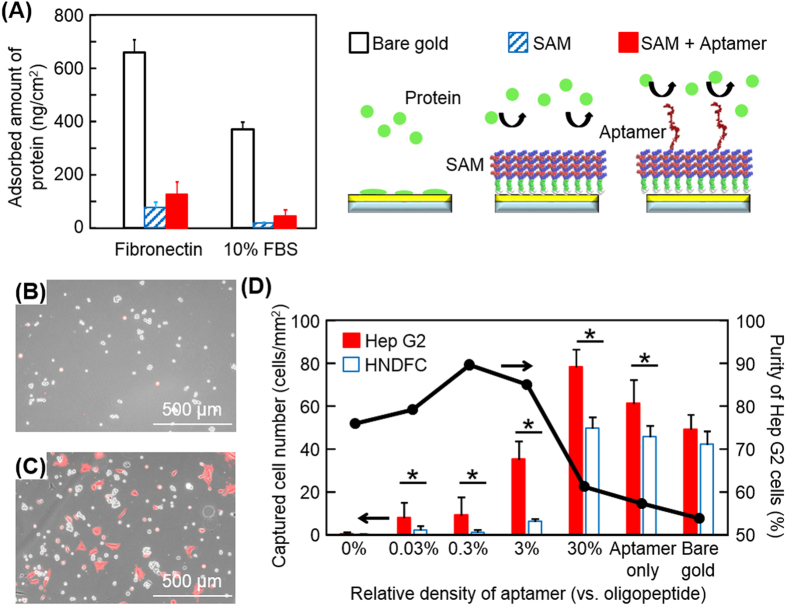
Non-specific protein adsorption and selective cell capture. (**A**) Protein-repulsive characteristics on oligopeptide SAMs. Bare gold surface (open column), the SAM surface (striped column), and the aptamer-conjugated SAM surface (solid column) were exposed to fibronectin and proteins in 10% FBS. The error bars indicate the standard deviations calculated from four independent experiments with QCM. (**B,C**) Hep G2 cells and RFP-HNDFCs captured on an aptamer-modified surface. Phase-contrast and fluorescent images were merged. Red fluorescence signal indicates RFP-HNDFCs. The two cell types were seeded at a ratio of 1:1 on the gold surfaces modified with (a) 3% aptamer/oligopeptide, or (b) without any modification. (**D**) Selective cell capture on SAMs modified with aptamer. A mixed suspension of Hep G2 cells (target, solid column) and HNDFCs (non-target, open column) was seeded on the aptamer-SAM surfaces of different aptamer densities. The relative density of aptamer represents the molar ratio of the aptamer to the oligopeptide calculated from QCM data. The error bars indicate the standard deviations calculated from nine independent experiments. *p < 0.05.

A hydrated and electrically neutral surface is low-fouling to the non-specific adsorption of proteins, and thus, cells. Zwitterionic polymers, such as poly(carboxybetaine) and poly(sulfobetaine), have been used to prepare anti-fouling surfaces^19^. Surfaces modified with zwitterionic oligopeptides, such as repeated lysine-glutamic acid (KE) or lysine-aspartic acid (KD) (i.e. EKEKEKE or DKDKDKD), also exhibit strong resistance to protein adsorption and cell adhesion^15^,^17^. In the present study, the oligopeptide, CGGGKEKEKEK, was designed to form a cell-repulsive SAM on a gold surface due to electrostatic forces generated by the alternating positive (K) and negative (E) amino acids between neighboring oligopeptides (Figure S1, Supporting Information)^17^. The aptamer was conjugated to the oligopeptide SAM with azide-alkyne cycloaddition through copper-catalyzed click chemistry (Fig. 1). The dependence of aptamer configurations on its density (1%, 2%, and 3% aptamer density with respect to the number of oligopeptides) was examined using MD calculations in implicit solvents. The aptamer shows striking differences in behavior in the three different conditions (Fig. 2B). At the lowest density (1%), the aptamer flattens on the oligopeptide SAM surface. Conversely, at the higher aptamer densities (2% and 3%), the aptamer is extended towards the solvent for 14.7 nm and 18.9 nm, respectively. These different tendencies can be explained by the fact that the increase in the density lowers the relative distance between negatively charged aptamers (~100 nm, 1%; ~33 nm, 3%), which repulse each other, preventing the aptamer from flattening on the peptide surface. The configurations of the aptamer may be responsible for its affinity to specific cells and such correlations were observed in experiments as discussed below (Fig. 3D).

### Experimental characterizations of aptamer-conjugated oligopeptide SAM

To conjugate the aptamer to the oligopeptide SAM using the azide-alkyne click chemistry reaction^20^,^21^, the alkyne was coupled to the hydroxyl group of 5′ thymine of the aptamer, using the alkyne-modifier serinol phosphoramidite (Glen Research, USA), and the NH_2_ of the K (Lys) at the C-terminus was replaced with an azide (-N_3_). The oligopeptides with and without azide groups (peptide-N_3_ and peptide-COOH) were mixed in an aqueous solution at a ratio of 1:9, of which drops were then placed on a gold-coated glass substrate. Because a thiol group containing cysteine spontaneously binds to gold through gold-thiolate bonding, the oligopeptides chemically adsorb and form a SAM on the gold substrate. We quantified the adsorption of the oligopeptides and subsequent conjugation of the aptamer with click chemistry using a quartz crystal microbalance (QCM). The adsorbed amounts of oligopeptides and aptamer were ~0.16 nmol/cm^2^ and ~4.7 pmol/cm^2^, respectively. These values indicate that the oligopeptides formed a dense molecular layer, which was sufficient to block non-specific adsorption of proteins (see below), and that the number of aptamers against oligopeptides was approximately 3:100 based on their molecular weights. QCM was further employed to examine the non-fouling properties of the oligopeptide SAM and the aptamer-conjugated SAM. In the measurements, the surfaces were exposed to culture media containing fibronectin (a cell-adhesive protein) and 10% fetal bovine serum (FBS, a mixture of various proteins). As shown in Fig. 3A, the adsorptions of both fibronectin and FBS proteins were significantly reduced on the oligopeptide SAM compared to those on a bare gold surface. However, the adsorption of fibronectin (75.3 ng/cm^2^) was much higher than that on a SAM composed of only peptide-COOH in our previous study (44.2 ng/cm^2^)^17^. This is likely because the addition of 10% peptide-N_3_ altered the balances of electrostatic and/or hydrophilic/hydrophobic conditions while the oligopeptide without azide groups was originally designed to form a SAM with electrically neutral and highly hydrophilic characteristics. The conjugation of the aptamer on the SAM further increased the adsorption of fibronectin and proteins in FBS probably because of its negative charge and hydrophobicity.

### Dependence of cell capture efficiency on mixing ratio of peptide-N_3_

The results from the MD simulations and QCM measurements indicate that the density of aptamers is closely involved in both the configuration of the aptamer (thus cell binding affinity) and non-specific protein adsorption. Since the aptamer density could be readily modulated by changing the mixing ratio of peptide-N_3_ to peptide-COOH, we examined the number of target cells captured and the target/non-target ratio on different aptamer-conjugated SAM surfaces when prepared with 0.1%, 1%, 10%, and 100% peptide-N_3_. Note that the aptamer density (aptamer/oligopeptide) was defined in this study based on the QCM results as 0.03%, 0.3%, 3%, and 30% when the mixing ratio of peptide-N_3_ were 0.1%, 1%, 10%, and 100%, respectively. As controls, a bare gold surface (bare gold) and a surface without oligopeptide layer (aptamer only) was examined. A cell suspension mixture containing Hep G2 cells (targets, 1 × 10^5^ cells/2 mL) and red fluorescent protein expressing human neonatal dermal fibroblast cells (RFP-HNDFCs, non-targets, 1 × 10^5^ cells/2 mL) were seeded on the aptamer-conjugated SAM surfaces in a typical 6-well culture plate. After 3 h of culture, phase-contrast and fluorescent microscopic images were taken and the number of each cell type was counted (Fig. 3B and C). The resultant target/non-target ratios were improved on the surfaces of 0.03%, 0.3%, and 3% aptamer/oligopeptide and no significant difference was observed between them (Fig. 3D). However, the total number of captured Hep G2 cells in 3% aptamer/oligopeptide was three to four times greater than those on 0.03% and 0.3% aptamer/oligopeptide and reached ~70% of that on bare gold. These are possibly consistent with the results obtained with MD simulations, where the aptamer flattens on the SAM in <1% aptamer/oligopeptide but is exposed toward bulk at >2% aptamer/oligopeptide. On 30% aptamer/oligopeptide, the number of attached Hep G2 cells increased and the capture efficiency reached 35%, which was greater than that on the 3% aptamer/oligopeptide surface (~20%). However, adhesion of HNDFCs also significantly increased and the purity dropped from 85% on the 3% aptamer/oligopeptide surface to 61% on the 30% aptamer/oligopeptide surface. This was probably because the net surface charge became negative due to the increased number of aptamers. Based on these results, we used 3% aptamer/oligopeptide in the following experiments.

### Harvesting captured cells using electrochemistry

**Figure 4:**
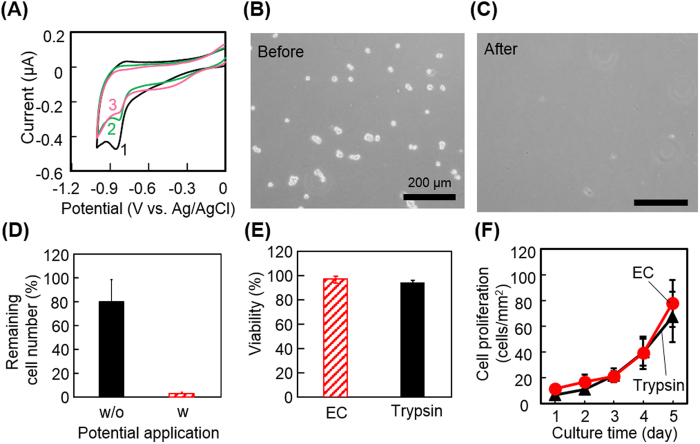
Characterizations of electrochemical cell detachment. (**A**) Cyclic voltammograms during reductive desorption of the peptide-aptamer layer. The digits 1, 2, and 3 indicate the scan numbers. The scanning rate was 20 mV/s. (**B,C**) Phase-contrast images of Hep G2 cells on a gold surface before and after the application of −1.0 V with respect to Ag/AgCl. (**D**) Quantitative analysis of the electrochemical cell detachment. The number of cells was counted after conducting entire procedures for the detachment with (w, striped column) and without (w/o, solid column) the potential application. (**E**) Viability of cells detached by electrochemical potential application (EC, striped column) or by treatment using trypsin (solid column). (**F**) Proliferation of electrochemically detached cells. Cells were detached by electrochemical potential application (EC, closed circle) or by treatment using trypsin (closed triangle) and re-seeded in a conventional culture dish. Changes in the number of cells were counted. (**D–F**) The values and error bars represent means and standard deviations calculated from three independent experiments.

The gold-thiolate bond can be reductively cleaved by applying a negative electrochemical potential. With this reaction, we previously demonstrated that cells attached on alkanethiol or oligopeptide SAMs were detached along with the desorption of SAMs^17^,^22^,^23^,^24^,^25^. Here we examined whether using the same electrochemical reaction, the aptamer-conjugated oligopeptide SAM is cleavable and whether captured cells can be collected. Cyclic voltammetry was employed to determine the potential required for the reductive desorption. The results showed that the peak potential appeared at −0.87 V with respect to Ag/AgCl in the first scan, which decayed in the second and third scans, showing that the gold-thiolate bond of the aptamer-conjugated SAM was cleaved at this potential (Fig. 4A). It was reported with alkanethiol SAMs that the peak potential was associated with the stability of SAMs and became more negative in SAMs with longer alkyl chains^26^,^27^. Because the peak potential of the aptamer-conjugated SAM was almost the same as that of the oligopeptide SAM, the mixing of the azide-modified oligopeptide and conjugation with the aptamer may not be involved in the stability. We decided to use −1.0 V with respect to Ag/AgCl, a negative potential that is sufficiently greater than the peak, to harvest cells in the subsequent experiments because the cell membrane could be electrically resistant and cause a potential drop. As expected, Hep G2 cells captured on the aptamer-conjugated SAM were detached from the surface by applying −1.0 V with respect to Ag/AgCl for 5 min (Fig. 4B and C). Quantitative analysis of the detachment was carried out by counting the number of Hep G2 cells on the substrate before and after the potential application (Fig. 4D). More than 97% of the cells were detached with 5 min of potential application. Moreover, it is noteworthy that ~20% of captured cells were detached without potential application. This is probably because the control samples experienced the same procedures as did the samples with potential application, including removal of culture medium, washing with PBS, and placement onto the electrode system (see the image in Fig. 1). To check the viable conditions of the cells collected with the electrochemical reaction, live/dead fluorescent staining (Fig. 4E) and proliferation assay (Fig. 4F) were conducted. There was no significant difference in the viability and proliferation rate between the cells collected with the electrochemical reaction and with a typical trypsin treatment, showing that electrochemical detachment is a non-cytotoxic and cell-friendly approach on at least the same level as the optimized trypsin treatment. This rapid and non-cytotoxic cell detachment approach could be a great advantage compared with other approaches using cDNA or endonuclease (10–30 min), particularly when the processes are repeated to obtain a high purity of cell population^28^,^29^,^30^.

### Liver-specific functions of enriched Hep G2 cells

**Figure 5:**
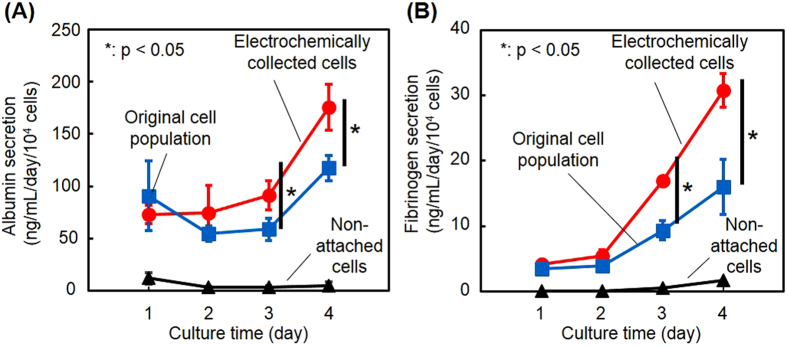
Liver-specific functions of electrochemically detached cells. The secretions of albumin (**A**) and fibrinogen (**B**) were compared in cell populations in three cultures: cells enriched on the aptamer-SAM and collected by electrochemical detachment, cells that did not attach on the aptamer-SAM surface, and cells before separation (Hep G2 and HNDFCs were equally mixed). The values and error bars represent mean and standard deviations calculated from three independent experiments. *p < 0.05.

The hepatic functions of Hep G2 cells were evaluated after they were enriched from a mixture with HNDFCs on the aptamer-conjugated SAM, collected with the electrochemical reaction, and seeded at 3 × 10^4^ cells/well on a conventional 24-well plate. For comparison, cells that had not been captured on the SAM in the 3-h incubation were collected and seeded at the same cell density on a 24-well plate. Further, the original cell suspension before the enrichment containing an equal number of Hep G2 cells and HNDFCs was seeded on a 24-well plate at the same cell density. Culture media in all three cultures were sampled every 24 h over 4 days and the concentrations of albumin and fibrinogen were determined. At days 1 and 2, there was no significant difference in the secretion of both proteins between the cultures with or without cell enrichment, while thereafter the effects of the enrichment became apparent (Fig. 5). Although co-culture of hepatocytes with fibroblasts typically upregulates hepatic functions including albumin secretion^31^, Hep G2 cells is derived from hepatoblastoma and could be less sensitive to surrounding cells and microenvironments. The results are more likely attributed to the proliferation of Hep G2 cells.

### Multiple enrichment of target cells

**Figure 6:**
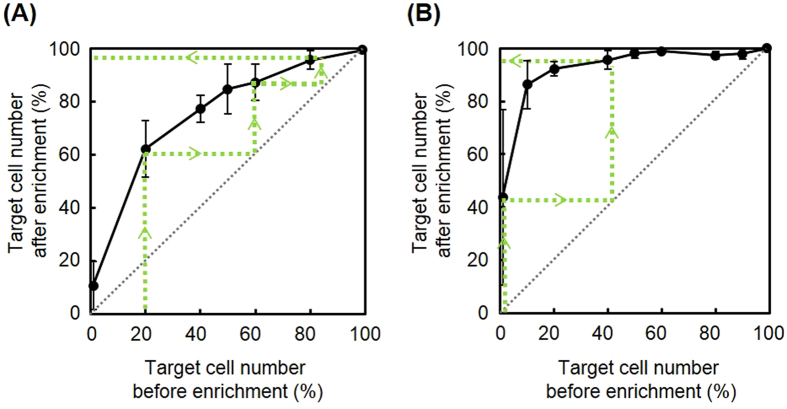
Separation curve of target cells. (**A**) Ratio of Hep G2 (targets) to HNDFCs or (**B**) ratio of Hep G2 (targets) to normal human hepatocytes before and after separation using the aptamer-SAM. Broken lines with arrows indicate a theoretical example of multistep enrichment. The values and error bars indicate mean and standard deviations calculated from three independent experiments.

The aptamer-conjugated SAM surface was useful in improving the purity of Hep G2 cells out of HNDFCs from 50% to 85%. However, this value can be insufficient depending on the intended uses or applications. Microfluidics could be a promising solution for this because cell isolation under flow shear stress potentially provides an optimum threshold condition for selective cell capture^32^, facilitating cell separation on a denser aptamer, and thus, higher cell affinity surfaces. However, a drawback of microfluidic approaches is the limitation on the number of cells that can be processed at once, which makes the system more complicated and expensive as seen in fluorescent-activated cell sorting. This in turn compromises the benefits gained from aptamers such as inexpensive, rapid acquisition and scalability. A more straightforward way may be repeating the enrichment processes using a flat plate. To obtain a separation curve for calculating the number of theoretical stages, we prepared cell suspensions with the same total cell number (2 × 10^5^ cells /2 mL/well) but different ratios of Hep G2 cells to HNDFCs (Hep G2: 1%, 20%, 40%, 50%, 60%, 80%, and 99%). The cell suspensions were exposed to the aptamer-conjugated SAM surface for 3 h and obtained data were plotted in Fig. 6A. Note that the x-axis and y-axis in Fig. 6A indicate the purity before and after the enrichment, respectively. An example of prospective changes in the purity through multiple enrichments is shown by the broken lines with arrows in Fig. 6A, representing that triple repetition of the enrichment increases the purity from 20% to 97%.

Enrichment of specific cancer cells from normal cells may be an interesting and important application of this approach. The same enrichment experiments were conducted using normal human primary hepatocytes instead of HNDFCs. As shown in Fig. 6B, much more efficient enrichment of Hep G2 cells was observed against normal human hepatocytes than in those purified against HNDFCs. Only through two repetitions of the enrichment did the purity increase from 1% to >95%. This is attributed to the fact that the aptamer was originally designed by means of cell-SELEX using human normal hepatocytes as counterparts. On the other hand, the results also indicate the possible limitation that a small amount of non-target cells was unexclusive and it may be challenging to obtain pure cell populations only by repeating the enrichment processes. Furthermore, although a sample containing only 1% of the target cells (2000 cells/mL) could be treated and enriched on the modified surface, the error bars were relatively large compared to the number of cells obtained. This implies that cell isolation of a sample with a cell concentration less than 2000 cells/mL would be challenging. Further optimisation of peptide sequences and isolation times will be necessary to improve this cell isolation limitation. In particular, the peptide sequence should be adjusted to make the surface electrically neutral when the aptamer is bonded to prevent any non-specific cell adhesion, potentially making it possible to expose cells to the aptamer surface for longer periods of time.

We previously demonstrated that electrochemical cell detachment could be conducted at single-cell resolution in a specially controlled manner^33^. On an array of microelectrodes, single cells at specific areas under a microscope were selectively detached on demand by applying a potential to specific microelectrodes under the target cells. A combination of the aptamer-conjugated SAM and a microelectrode array could be beneficial to select cells with interesting features under a microscope and collect or exclude them for following analysis.

## Conclusions

This study describes a cell isolation platform developed using the combination of selective cell attachment with an aptamer and non-cytotoxic cell detachment with electrochemistry. An aptamer-conjugated surface was designed based on molecular dynamics calculations of aptamers on an oligopeptide SAM and experiments using QCM. The aptamer-conjugated oligopeptide SAM sufficiently decreased non-specific protein adsorption and was beneficial for specifically attracting hepatic cancer cells from other cell types. The cells captured on the aptamer-conjugated SAM surface were detached by potential application for 5 min. The collected cells maintained high viability, proliferation activity, and liver-specific functions comparable to those of cells treated with trypsin. Although further studies on the repetitions of the processes in a precisely controlled manner and the combinations with microelectrode arrays are required, this non-cytotoxic and versatile cell isolation approach could be a useful platform in various fields including basic cancer research and regenerative medicine.

## Methods

### Cell preparation

Human hepatic cancer cells (Hep G2 cells; RCB1642, Riken Cell Bank, Japan), red fluorescent protein-expressing human neonatal dermal fibroblasts (RFP-HNDFCs; cAP-0008RFP, Angio-Proteomie, USA), and normal human hepatocytes (SA152, KaLy-Cell, France) were purchased. Hep G2 cells were maintained in Dulbecco’s modified Eagle’s medium (DMEM; Sigma-Aldrich, Japan) supplemented with 10% fetal bovine serum (FBS; Sigma-Aldrich, Japan) and 1% penicillin/streptomycin (Life Technologies, USA). RFP-HNDFCs were maintained in minimum essential medium α (Thermo Fisher, Japan) supplemented with 10% FBS and 1% penicillin/streptomycin. The culture media were exchanged every two days and the cells were passaged with 0.25% trypsin-EDTA (Thermo Fisher, Japan). When Hep G2 cells were mixed with the other types of cells (HNDFCs or normal hepatocytes), we used the culture medium for Hep G2 cells.

### Modification of oligopeptides and aptamer on a gold substrate

A glass substrate (24 mm × 24 mm, No. 4, Matsunami, Japan) was sputter-coated with a few nanometers of chromium and ~40 nm of gold. The oligopeptide, CGGGKEKEKEK (peptide-COOH; Scrum, Japan), was designed to form a cell-repulsive layer on the gold substrate^15^,^17^. Its azide-modified form CGGGKEKEKEK-azide (peptide-N_3_; Toray, Japan) was also synthesized. A mixed aqueous solution of these two oligopeptides was prepared at different ratios but at a fixed total concentration (50 μM). The gold-coated substrates were immersed into the oligopeptide mixture solutions overnight at 4 °C. Since the oligopeptides contain cysteine at the N-terminal and the alternating zwitterionic KE sequence at the C-terminal, the oligopeptides chemically adsorb onto the gold substrate via gold-thiol bonding and form a dense self-assembled monolayer (SAM) by electrostatic interactions. After washing with phosphate-buffered saline (PBS), the layer was further modified with an aptamer, 5′-alkyne-TAACTCAATAAGCTAGGTGGGTGGGGGACACTACTCGGGGGTGGTTGGGT-3′ (BEX, Japan) through azide-alkyne click chemistry^20^,^21^ by placing a drop of 0.3 μM aptamer solution (H_2_O:t-BuOH, 2:1) containing 1.8 mol% CuSO_4_ and 8 mol% sodium ascorbate on the substrate and incubating for 8 h at room temperature. Note that the aptamer sequence was previously designed using cell-SELEX for the selection of Hep G2 cells from normal human hepatocytes^18^. The substrate was then rinsed with PBS and sterilized with 70% ethanol. Each substrate was then placed in a 6-well plate (BD Falcon, Japan).

### Simulation of oligopeptide and aptamer behaviors

MD simulations were carried out using software NAMD2.9^34^ with CHARMM22^35^ and GolP force field^36^, and CMAP correction^37^. Golp-FF force field describes the interaction between Au(111) and proteins. The pdb files of the aptamer, azide-alkyne linker, and oligopeptide SAM were designed with the software Pymol^38^, while the gold unit slab file was available with GolP force field. All analyses were carried out with VMD software^39^. We model the aptamer in explicit and implicit solvents in order to test the impact of the implicit approximation, which is necessary for the reduction of calculation volume of the aptamer-conjugated SAM. The implicit solvent is modeled with the Generalized Born Implicit Solvent. For the explicit solvent, the aptamer was solvated in a water box (109 Å × 234 Å × 128 Å) and neutralized with the addition of Na^+^- Cl^−^ ions at physiological concentration (0.15 mol/L). A preliminary minimization of 100,000 steps was carried out. Subsequently, MD simulation was carried out for a simulation time of 8 ns at a temperature of 310 K with a time step of 2 fs. The explicit solvent simulation was run under constant pressure and temperature (NPT) conditions (pressure 1 atm, temperature 310 K). The SAM was built at the density obtained from experimental data with QCM. We modeled three different aptamer densities: ~1% (1 aptamer every 108 peptides), ~2% (1 aptamer every 56 peptides) and ~3% (1 aptamer every 36 peptides). Aptamer-conjugated SAM systems are simulated in implicit solvent with the Generalized Born Implicit Solvent. All systems were minimized with the steepest descent algorithm for at least 15,000 steps. Subsequently, MD simulations were run until structural stability (monitored by root mean square deviation) for a total time of 10–15 ns.

### QCM

QCM (AFFINIX QN, Initium, Japan) was used to quantify the stepwise loading of molecules on a gold substrate: adsorption of the oligopeptides on the gold surface, binding of the aptamer on the SAM, and non-specific adsorption of proteins on the aptamer-conjugated SAM. Briefly, a gold QCM electrode was cleaned with piranha solution (H_2_SO_4_:H_2_O_2_, 3:1), rinsed with double-distilled water (ddH2O; Milli-Q Advantage, Millipore-Japan, Japan), and dried under nitrogen gas. The electrode was immersed in a peptide mixture solution (peptide-COOH:peptide-N_3_, 9:1; 50 μM) overnight at 4 °C, and then rinsed with ddH_2_O and dried under nitrogen gas. The change in resonance frequency before and after the modification in air was measured. The electrode was then immersed into a 0.3 μM aptamer solution and incubated for 8 h at room temperature. The electrode was then rinsed with ddH_2_O and dried, and the change in resonance frequency was measured. In the examinations of protein adsorption, the electrodes covered with the aptamer-conjugated SAM were exposed to a 1 mg/mL fibronectin (Sigma-Aldrich, Japan) solution or culture medium containing 10% FBS for 1 h at room temperature. Changes in resonance frequency before and after the exposures were measured in dried condition. The frequency differences were translated to adsorbed amounts using the Sauerbrey equation. An unmodified gold electrode was used as a reference surface.

### Selective capture of target cells

Hep G2 cells and RFP-HNDFCs were mixed at 1.0 × 10^5^ cells per cell type in 2 mL of culture medium. Aptamer-conjugated oligopeptide SAMs were prepared using mixtures of peptide-N_3_ and peptide-COOH at 0.1%, 1%, and 10% peptide-N_3_. The cell mixture was poured on the aptamer-conjugated SAMs. After 3 h of culture, non-attached cells were washed twice with PBS and the number of each type of cells was counted on microscopic images using ImageJ software. SAMs without conjugation of aptamers and bare gold surfaces were used as controls. To obtain a separation curve through multistep enrichment of target cells, separation with the oligopeptide SAM was examined using cell suspensions at various ratios of Hep G2 cells to RFP-HNDFCs or normal human hepatocytes. In both cases, the cell density was 2.0 × 10^5^ cells/2 mL/well. To distinguish Hep G2 cells from normal human hepatocytes, Hep G2 cells were previously stained with Vybrant-Dil (Thermo Fisher, Japan) in DMEM without serum for 15 min at 37 °C, followed by washing thrice with DMEM supplemented with 10% FBS. The number of fluorescently labeled and non-labeled cells was counted on microscopic images.

### Potential for reductive desorption of SAM

Cyclic voltammetry was employed to determine the potential required for the reductive desorption of the aptamer-conjugated SAM from the gold surface. The modified gold substrate, a Ag/AgCl reference electrode, and a platinum auxiliary electrode were connected to an electrochemical measurement system (Metrohm Autolab, The Netherlands). Cyclic voltammograms were taken with the three-electrode configuration in a 0.5 M KOH solution previously deoxygenated by bubbling nitrogen gas for 20 min. Scanning was conducted three times at 20 mV/s in the range from 0 to −1.0 V. All potential values refer to those measured with respect to the Ag/AgCl electrode.

### Electrochemical cell detachment

In the evaluation of detachment of cells, a hand-built culture chamber (volume, 1 mL; Fig. 1B) was fabricated to facilitate cell seeding and potential application on the substrate. The chamber consisted of a cylindrical wall of polydimethylsiloxane (PDMS; ShinEtsu Chemical, Japan), the gold-coated glass substrate (40 mm × 50 mm, Matsunami, Japan), an iron ring, and eight neodymium magnet blocks (φ3.0 mm, Magfin, Japan). The magnet blocks were embedded in the PDMS so that the gold substrate was pinched between the PDMS and the iron ring by magnetic forces. The gold substrate was modified with the oligopeptides (peptide-COOH: peptide-N_3_, 9:1) and the aptamer as described above. Hep G2 cells were seeded in the culture chamber at a density of 1.0 × 10^5^ cells/2 ml/well and cultured for 3 h. After the culture surface was rinsed twice with PBS, the gold substrate and an Ag/AgCl reference electrode were connected to a potentiostat (HA-151, Hokuto Denko, Japan). After applying −1.0 V for 5 min and gently rinsing with PBS, phase-contrast microscopic images were acquired to quantify the remaining cells. Control experiments were conducted in the same manner but without potential application. Detached cells were collected to examine cell viability using live/dead fluorescent staining with fluorescein diacetate and ethidium bromide. In the evaluation of proliferation activity, detached cells were re-seeded in a conventional 24-well plate at 1 × 10^4^ cells/500 μL/well and cultured for 5 days. Phase-contrast images were obtained every 24 h to quantify the growth rate of cells. The growth rate was compared with that of cells detached using 0.25% trypsin-EDTA.

### Evaluation of liver-specific functions

To examine the influence of cell detachment and enrichment on cell functions, the secretion of two liver-specific proteins, albumin and fibrinogen, was measured after capture and electrochemical detachment using the aptamer-conjugated SAM. In the evaluations, a mixed cell suspension of Hep G2 cells and HNDFCs (1.0 × 10^5^ cells/mL for each cell line) was seeded on the aptamer-conjugated SAM (3% aptamer density) and cultured for 3 h. Cells that remained attached on the SAM surface were detached electrochemically as described above and re-seeded in a 24-well culture plate at 3.0 × 10^4^ cells/500 μL/well. Cells that did not attach on the SAM surface were collected and re-seeded in a 24-well plate at the same cell density for comparison. The original suspension of Hep G2 cells and HNDFCs (1.0 × 10^5^ cells/mL for each cell line) was seeded in a 24-well culture plate at the same cell density as another control. Culture media in these three systems were sampled every 24 h. The amounts of albumin and fibrinogen in the samples were analyzed using sandwich ELISA assay kits according to the manufacturer’s instructions (Abcam, Japan).

### Data analysis

The number of experiments is described in the figure captions. Numerical variables were statistically evaluated by Student’s t-test for Figs 3 and 5. A value of p < 0.05 was considered significant.

## Additional Information

**How to cite this article:** Enomoto, J. *et al*. Catch-and-Release of Target Cells Using Aptamer-Conjugated Electroactive Zwitterionic Oligopeptide SAM. *Sci. Rep.* **7**, 43375; doi: 10.1038/srep43375 (2017).

**Publisher's note:** Springer Nature remains neutral with regard to jurisdictional claims in published maps and institutional affiliations.

## Supplementary Material

Supplementary Information

## References

[b1] Yu M., Stott S., Toner M., Maheswaran S. & Haber D. A, Circulating tumor cells: approaches to isolation and characterization. J. Cell Biol. 192, 373–382 (2011). 21300848 10.1083/jcb.201010021 PMC3101098

[b2] Damm G. . Human parenchymal and non-parenchymal liver cell isolation, culture and characterization. Hepatol. Int. 7, 951–958 (2013). 26202025 10.1007/s12072-013-9475-7

[b3] Diogo M. M., da Silva C. L. & Cabral J. M. Separation technologies for stem cell bioprocessing. Biotechnol. Bioeng. 109, 2699–2709 (2012). 22887094 10.1002/bit.24706

[b4] Radisic M., Iyer R. K. & Murthy S. K. Micro-and nanotechnology in cell separation. Int. J. Nanomedicine. 1, 3 (2006). 17722258 10.2147/nano.2006.1.1.3 PMC2426772

[b5] Ellington A. D. & Szostak J. W. *In vitro* selection of RNA molecules that bind specific ligands. Nature. 346, 818–822 (1990). 1697402 10.1038/346818a0

[b6] Galina S. Z. . Electrochemical aptasensor for lung cancer-related protein detection in crude blood plasma samples. Sci. Rep. 6, 34350 (2016). 27694916 10.1038/srep34350 PMC5046130

[b7] Guo K.-T., Paul A., Schichor C., Ziemer G. & Wendel H. P. Cell-SELEX: Novel perspectives of aptamer-based therapeutics. Int. J. Mol. Sci. 9, 668–678 (2008). 19325777 10.3390/ijms9040668 PMC2635693

[b8] Marangoni K. . Prostate-specific RNA aptamer: promising nucleic acid antibody-like cancer detection. Sci. Rep. 5 (2015). 10.1038/srep12090 PMC4502603 26174796

[b9] Kim J. . Integrated Microfluidic Isolation of Aptamers Using Electrophoretic Oligonucleotide Manipulation. Sci. Rep. 6 (2016). 10.1038/srep26139 PMC4877600 27217242

[b10] Saito S. . Rapid acquisition of high-affinity DNA aptamer motifs recognizing microbial cell surfaces using polymer-enhanced capillary transient isotachophoresis. Chem. Commun (2016). 10.1039/c5cc07268a 26525483

[b11] Fang X. & Tan W. Aptamers generated from cell-SELEX for molecular medicine: a chemical biology approach. Acc. Chem. Res. 43, 48–57 (2009). 10.1021/ar900101s PMC2808443 19751057

[b12] Kim Y., Liu C. & Tan W. Aptamers generated by Cell SELEX for biomarker discovery. Biomark. Med. 3, 193–202 (2009). 20477510 10.2217/bmm.09.5

[b13] Zhang Z., Chen N., Li S., Battig M. R. & Wang Y. Programmable hydrogels for controlled cell catch and release using hybridized aptamers and complementary sequences. J. Am. Chem. Soc. 134, 15716–15719 (2012). 22970862 10.1021/ja307717w

[b14] Feng L., Li W., Ren J. & Qu X. Electrochemically and DNA-triggered cell release from ferrocene/β-cyclodextrin and aptamer modified dualfunctionalized graphene substrate. Nano Res. 8, 887–899 (2015).

[b15] Chen S., Cao Z. & Jiang S. Ultra-low fouling peptide surfaces derived from natural amino acids. Biomaterials. 30, 5892–5896 (2009). 19631374 10.1016/j.biomaterials.2009.07.001

[b16] Yeo W.-S. & Mrksich M. Electroactive self-assembled monolayers that permit orthogonal control over the adhesion of cells to patterned substrates. Langmuir. 22, 10816–10820 (2006). 17129065 10.1021/la061212y PMC2536489

[b17] Kakegawa T., Mochizuki N., Sadr N., Suzuki H. & Fukuda J. Cell-adhesive and cell-repulsive zwitterionic oligopeptides for micropatterning and rapid electrochemical detachment of cells. Tissue Eng Part A. 19, 290–298 (2013). 22853640 10.1089/ten.tea.2011.0739 PMC3530950

[b18] Ninomiya K. . Cell-SELEX based selection and characterization of DNA aptamer recognizing human hepatocarcinoma. Bioorg. Med. Chem. Lett. 23, 1797–1802 (2013). 23403083 10.1016/j.bmcl.2013.01.040

[b19] Ladd J., Zhang Z., Chen S., Hower J. C. & Jiang S. Zwitterionic polymers exhibiting high resistance to nonspecific protein adsorption from human serum and plasma. Biomacromolecules. 9, 1357–1361 (2008). 18376858 10.1021/bm701301s

[b20] Hein J. E. & Fokin V. V. Copper-catalyzed azide-alkyne cycloaddition (CuAAC) and beyond: new reactivity of copper(I) acetylides. Chem. Soc. Rev. 39, 1302–1315 (2010). 20309487 10.1039/b904091a PMC3073167

[b21] Spiteri C. & Moses J. E. Copper-catalyzed azide-alkyne cycloaddition: regioselective synthesis of 1,4,5-trisubstituted 1,2,3-triazoles. Angew. Chem. Int. Ed. Engl. 49, 31–33 (2010). 19921729 10.1002/anie.200905322

[b22] Mochizuki N. . Tissue engineering based on electrochemical desorption of an RGD-containing oligopeptide. J. Tissue Eng. Regen. Med. 7, 236–243 (2011). 22162306 10.1002/term.519

[b23] Kageyama T. . Rapid engineering of endothelial cell-lined vascular-like structures in *in situ* crosslinkable hydrogels. Biofabrication. 6, 025006 (2014). 24658207 10.1088/1758-5082/6/2/025006

[b24] Osaki T. . Acceleration of vascular sprouting from fabricated perfusable vascular-like structures. PLoS One. 10, e0123735 (2015). 25860890 10.1371/journal.pone.0123735 PMC4393106

[b25] Enomoto J. . Engineering thick cell sheets by electrochemical desorption of oligopeptides on membrane substrates. Regenerative Therapy. 3, 24–31 (2016). 10.1016/j.reth.2015.12.003 PMC6581802 31245469

[b26] Inaba R., Khademhosseini A., Suzuki H. & Fukuda J. Electrochemical desorption of self-assembled monolayers for engineering cellular tissues. Biomaterials. 30, 3573–3579 (2009). 19362363 10.1016/j.biomaterials.2009.03.045

[b27] Imabayashi S.-i. . Reductive desorption of carboxylic-acid-terminated alkanethiol monolayers from Au (111) surfaces. J. Electroanal. Chem. 428, 33–38 (1997).

[b28] Gaddes E. R. . Aptamer-based polyvalent ligands for regulated cell attachment on the hydrogel surface. Biomacromolecules. 16, 1382–1389 (2015). 25789558 10.1021/acs.biomac.5b00165

[b29] Li S., Chen N., Zhang Z. & Wang Y. Endonuclease-responsive aptamer-functionalized hydrogel coating for sequential catch and release of cancer cells. Biomaterials. 34, 460–469 (2013). 23083933 10.1016/j.biomaterials.2012.09.040

[b30] Zhao W. . Bioinspired multivalent DNA network for capture and release of cells. Proc. Natl. Acad. Sci. USA. 109, 19626–19631 (2012). 23150586 10.1073/pnas.1211234109 PMC3511714

[b31] Bhatia S., Balis U., Yarmush M. & Toner M. Microfabrication of Hepatocyte/Fibroblast Co‐cultures: Role of Homotypic Cell Interactions. Biotechnol. Prog. 14, 378–387 (1998). 9622518 10.1021/bp980036j

[b32] Plouffe B. D., Radisic M. & Murthy S. K. Microfluidic depletion of endothelial cells, smooth muscle cells, and fibroblasts from heterogeneous suspensions. Lab Chip. 8, 462–472 (2008). 18305866 10.1039/b715707j

[b33] Fukuda J., Kameoka Y. & Suzuki H. Spatio-temporal detachment of single cells using microarrayed transparent electrodes. Biomaterials. 32, 6663–6669 (2011). 21665269 10.1016/j.biomaterials.2011.05.068

[b34] Phillips J. C. . Scalable molecular dynamics with NAMD. J. Comput. Chem. 26, 1781–1802 (2005). 16222654 10.1002/jcc.20289 PMC2486339

[b35] Brooks B. R. . CHARMM: the biomolecular simulation program. J. Comput. Chem. 30, 1545–1614 (2009). 19444816 10.1002/jcc.21287 PMC2810661

[b36] Iori F., Di Felice R., Molinari E. & Corni S. GolP: An atomistic force‐field to describe the interaction of proteins with Au (111) surfaces in water. J. Comput. Chem. 30, 1465–1476 (2009). 19037859 10.1002/jcc.21165

[b37] Buck M., Bouguet-Bonnet S., Pastor R. W. & MacKerell A. D. Importance of the CMAP correction to the CHARMM22 protein force field: dynamics of hen lysozyme. Biophys. J. 90, L36–L38 (2006). 16361340 10.1529/biophysj.105.078154 PMC1367299

[b38] Delano W. L. The PyMOL Molecular Graphics System. Version 1 8 (2002).

[b39] Humphrey W., Dalke A. & Schulten K. VMD: visual molecular dynamics. J. Mol. Graph. 14, 33–38 (1996). 8744570 10.1016/0263-7855(96)00018-5

