# Supplementary material for: Catch-and-Release of Target Cells Using Aptamer-Conjugated Electroactive Zwitterionic Oligopeptide SAM
Source: Sci Rep. 2017 Mar 7;7:43375. doi: 10.1038/srep43375 (PMC5339905; doi:10.1038/srep43375)
Supplement: Supplementary Information [file srep43375-s1.pdf]

**Supplementary Information**

# Catch-and-Release of Target Cells Using Aptamer-Conjugated Electroactive Zwitterionic Oligopeptide SAM

Junko Enomoto[1], Tatsuto Kageyama[1], Tatsuya Osaki[1], Flavia Bonalumi[2], Francesca Marchese[2], Alfonso Gautieri[2], Elena Bianchi[3], Gabriele Dubini[3], Chiara Arrigoni[4], Matteo Moretti[4, 5, 6, 7], and Junji Fukuda[1]*

[1]Graduate School of Engineering, Yokohama National University, Japan

[2]Department of Electronics, Politecnico di Milano, Italy

[3]Department of Chemistry, Politecnico di Milan, Italy

[4]Cell and Tissue Engineering Laboratory, IRCCS Galeazzi Orthopaedic Institute, Italy

[5]Regenerative Medicine Technologies Lab, Ente Ospedaliero Cantonale, Switzerland

[6]Swiss Institute for Regenerative Medicine, Switzerland

[7]Cardiocentro Ticino, Switzerland

Correspondence and requests for materials should be addressed to J.F. (email: fukuda@ynu.ac.jp)

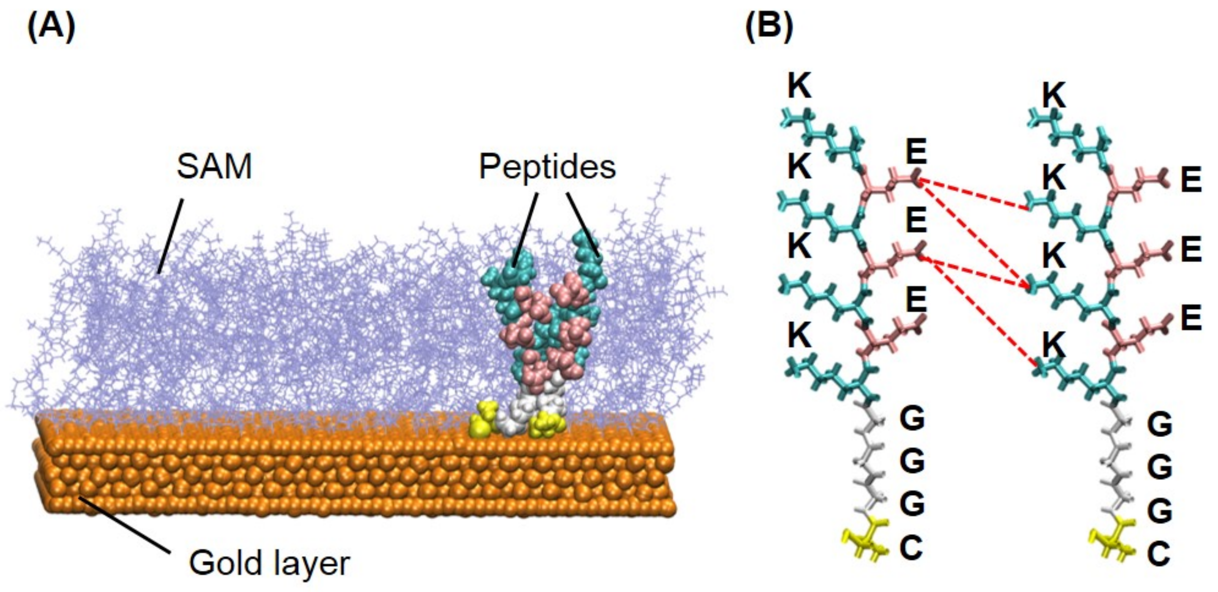


**Figure S1.** Dominant hydrogen bonds in self-assembly of oligopeptides. (A) SAM of oligopeptide-COOH after equilibration in explicit solvent. Two oligopeptides were highlighted with van der Waals representation. (B) Schematics of dominant interactions between two neighboring oligopeptides. The dashed lines represent major hydrogen bonds, indicating that the inter-peptide interactions are driving forces responsible for the formation of a dense molecular layer.
